# Failure to treat: an American policy perspective

**DOI:** 10.1017/S1092852924000543

**Published:** 2024-10-28

**Authors:** Katherine Warburton

**Affiliations:** 1 California Department of State Hospitals, Sacramento, CA, USA; 2 University of California Davis, Division of Psychiatry and the Law, Sacramento, CA, USA

**Keywords:** forensic psychiatry, public policy, criminal justice, mental health systems, international policy

## Abstract

Throughout its two and a half centuries in existence, US mental health policy has repeatedly failed people living with schizophrenia. The failures are cyclical—the inhumane conditions uncovered in the first 75 years of existence were addressed with the construction of state asylums to deliver moral treatment. One hundred years later, the asylums were themselves revealed to be inhumane. Deinstitutionalization, the response to the failure of asylums starting in the 1960s, now drives outcomes such as homelessness, incarceration, and early death for people living with psychotic illnesses. In all cases, well-intentioned policy reform has failed at the level of implementation, largely due to a lack of accountability. The result has been a consistent failure to adequately treat people living with schizophrenia, which is now understood to be a highly treatable condition. As the country passes into a quarter millennium in existence, reform is once again underway. Unlike other points in history, there is good news. Other countries, such as Italy, have successfully leveraged reform to achieve greatly improved outcomes. Understanding US history and the successful implementation of policy change in other countries is imperative and teaches us that accountability in implementation is necessary to break the cycle of policy failure.

## Introduction

The cyclical failure of mental health policy in the United States can be best articulated by those who contemporaneously documented it. In the 1840s, reformer Dorothea Linde Dix described the conditions of the insane to the Massachusetts Legislature with these words: “In cages, closets, cellars, stalls, pens! Chained, naked, beaten with rods, and lashed into obedience.” Dix’s subsequent efforts led to a vast expansion of moral treatment, delivered in bucolic state asylums throughout the United States.^1^^-^^4^ The proponents of the asylum movement expressed great confidence that the problem of injustice for the mentally ill had been solved.^5^

Roughly a century later, in 1951, journalist Albert Q. Maisel documented the now deteriorated conditions in Dix’s asylums in this manner: “We feed thousands a starvation diet […] we jam-pack men, women and sometimes even children into hundred-year-old firetraps in wards so crowded that the floors cannot be seen between the rickety cots, while thousands more sleep on ticks, on blankets or on the bare floors […] hundreds—of my own knowledge and sight—spend 24 hours a day in stark and filthy nakedness.”^6^ Media expose′s such as Maisel’s precipitated the deinstitutionalization movement.

Unfortunately, deinstitutionalization in the United States ultimately led to the transinstitutionalization of people with psychotic illness into jails and prisons. Just last year, in 2023, journalist Meg O’Connor gave an update on how people with mental illness were faring in the United States: “During the few hours that people with mental illness are allowed out of their cells […] they are shackled to tables. Some don’t have real clothing […] others smear feces on the walls of their cells. Flooded toilets are a regular occurrence. People scream and pace back and forth. Cells overflow with garbage. In certain housing units, mesh screens line the railings of the upper levels to prevent people from jumping.”^7^

The lack of progress is evident. What is less obvious are the factors driving the cyclical failure of well-intentioned policies. This policy history is complex, and the failures are multifactorial. Many accounts are reductive and fail to reflect the nuances inherent in large-scale policy reform. However, there is a common theme: a lack of accountability over the process of implementation.

## The first attempt

In the United States prior to the mid-1800s, people with severe psychotic disorders were generally chained, caged, and beaten. They were kept in squalid conditions in jails, in almshouses, or locked away in a family home. Within these conditions, people with psychotic disorders were often naked, cold, and/or in the dark. Their symptoms of hallucinations, delusions, and disorganization were attributed to moral failings or religious deviance, rather than illness.^8^^-^^10^

This situation began to change significantly in 1843, when Dorothea Dix wrote a report to the Massachusetts State Legislature documenting the misery she had observed upon touring the state. Dix had recently returned from England, where she met Samuel Tuke of the York Retreat. She was influenced by the advent of moral treatment championed by Philippe Pinel in France. The Massachusetts Legislature responded to her report and provided funding for improved conditions. Dix went on to replicate this approach in other states and was ultimately credited with the construction of over 30 asylums throughout the country.^1^^-^^4^

In the mid- to the late 1800s, the term “asylum” had a positive connotation. Mental Health asylums during this period most often resembled gothic castles built in the Kirkbride style, named for the psychiatrist-turned architect Thomas Story Kirkbride. These castles were installed on large, beautiful tracts of land. Here, patients could garden, exercise, and enjoy nature on self-sustaining campuses.^9^^-^^11^ An 1898 report from the asylum at Napa, California listed the copious amount of produce harvested from that campus as a key outcome for the year.^12^ Staff and patients lived together on campus. The treatment modality, known as moral treatment, was focused on kindness, compassion, and spiritual nourishment.

Moral treatment, and the asylums that delivered it, fell apart over the next century. Those castles championed by Dix grew overcrowded, understaffed, and neglected. A lack of judicial accountability in the form of vague involuntary detention laws led to unchecked growth. A lack of legislative accountability failed to fund infrastructure and resources to contend with that growth. No administrative accountability meant a lack of oversight provided to ensure humane conditions. The asylums became repositories for any person for whom society did not have a place. This dumping ground effect went beyond typical psychiatric illnesses and included such things as dependent elderly adults and patients with tertiary syphilis.^9^^,^ ^10^ In a Life magazine article titled *Bedlam 1946*, Albert Maisel reported, “thousands who might be restored to society linger in manmade hells for a release that comes more quickly only because death comes faster to the abused, the beaten, the drugged, the starved and the neglected.”^6^

Hence, the cruel, filthy, and inhumane conditions of the middle of the 1800s were manifest once again 100 years later. The descriptions by Maisel echoed those of Dix, and the origin of the problem was less about whether the patient was in the community or in an institution, and more about the lack of accountability for the humane treatment of patients with psychotic illness evidenced by the society. Conceptually, the reform of moral treatment in asylums was not the fundamental factor driving inhumane conditions. The blame lay in a lack of accountability and oversight.

## The second attempt

A federal response followed the state asylum failures. The National Mental Health Act was passed in 1943, which was quickly followed by the establishment of the National Institute of Mental Health (NIMH) in 1949. NIMH, and subsequently the Joint Commission on Mental Illness and Health, went to work exploring federally funded community alternatives to institutionalization. These efforts culminated in the Community Mental Health Act (CMHA) of 1963, which sought to provide federal funding for the resources needed to care for patients with psychosis in their communities. The creation of Medicaid and Medicare in 1965 provided federal funding.^10^^,^ ^13^^-^^16^

States slowly began to seize upon the opportunity to shift the cost of caring for this population to the federal government. Promising new treatments, such as the discovery of chlorpromazine, provided hope that this disease was treatable. The prohibitive potential costs of rehabilitating the state hospitals, combined with outrage at the civil liberties abuses resulting from indiscriminate institutionalization provided the political will to make change. These factors culminated in the process of deinstitutionalization, kicked off at the state level in California with the passage of the Lanterman-Petris-Short (LPS) Act. Lanterman was a conservative lawmaker concerned about the fiscal liability posed by the dilapidated state hospitals; Petris was a liberal lawmaker who was not convinced that schizophrenia was a real disease and who furthermore was intent upon restoring civil liberties to this population. It was a perfect political storm.^8^^,^ ^13^^-^^17^

The LPS act was signed into law in 1967 by California Governor Ronald Reagan and came into effect on July 1, 1969. This was the first state legislative attempt to limit psychiatric institutionalization. Referred to as the “Magna Carta” of mental health legislation, it facilitated deinstitutionalization by raising the bar for involuntary hospitalization to a very high “dangerousness standard.” To be involuntary hospitalized, a psychotic person had to be imminently dangerous to themselves or others, or so gravely disabled that they were essentially a danger to themselves. The nation followed suit, and the second round of mental health policy reform in the United States was well underway by the 1970s.^17^^,^ ^18^

Unfortunately, the federal dollars flowed into local governments without an accountability mechanism. There was no system in place to ensure program services would be developed specifically for deinstitutionalized people with psychotic illnesses. State governments, that continued to run the institutions, were effectively cut out of the funding stream. This created a problem that persists today, wherein the local recipient of federal funding lacks a fiscal incentive to prevent state institutionalization or incarceration. Additionally, the federal government ultimately failed to provide funding in the amount necessary to fully implement the CMHA. Local governments failed to focus the funding to support programs designed for deinstitutionalized persons.^15^ Rather, the money was used for patients with less severe conditions or was squandered altogether. In one case, for example, federal CMHA dollars were used to build athletic facilities.^13^ Community Mental Health Centers largely provided psychotherapy to middle-class patients without psychotic illnesses.^8^^,^ ^13^^,^ ^15^^,^ ^19^ This fractured and irresponsible system of funding systems resulted, once again, in those most in need falling through the cracks into homelessness and incarceration.^20^^-^^27^

A federal report noted in 1977 that “CMHCs attracted a new type of patient who was not very ill and was not a candidate for hospitalization in a state institution.”^22^^,^ ^27^ The precious federal dollars intended to support people with schizophrenia as they transitioned to community treatment were used to build tennis courts and swimming pools and hire lifeguards.^13^ The federal government was not held accountable to fund community programs in the manner promised, and the local governments were not held accountable to spend what money was coming in on the deinstitutionalized populations.

The result is that state institutions are now rapidly filling up with people suffering from schizophrenia, this time through a specific criminal commitment.

## Today: The incompetent to stand trial debacle

In 1960, the US Supreme Court held, in basic terms, that it is unconstitutional to try someone for a crime if they are too psychotic to understand trial processes or to rationally assist in their defense.^28^ Since deinstitutionalization, there has been an increasing trend of people with schizophrenia being arrested and found to be incompetent to stand trial (IST), which culminated following the Great Recession of 2009.^29^^-^^31^ The skyrocketing IST population has overwhelmed state hospitals, resulting in patients waiting excessive periods of time in jails to be admitted for restoration. This crisis, happening in most states today, has reached the ironic level of civil liberties groups suing states in federal court to institutionalize people for restoration, so that they may be sent to trial on criminal charges. An examination of the crisis illustrates the mechanics of implementation failure.

In 1972, a psychiatrist named Marc Abramson published a paper in the academic journal *Hospital and Community Psychiatry.* In it, he points out that the standard for involuntary treatment pre-LPS rested on the need for hospitalization, and post-LPS rested on dangerousness. This change resulted in a lack of consideration of psychiatric assessment for the latter standard. He noted a 100% increase in patients found IST in his county the year following the implementation of the LPS Act. He ends the paper by postulating that, “It would indeed be ironic if the Magna Carta of the mentally ill in California led to their criminal stigmatization and incarceration in jails and prisons, where little or no mental health treatment is provided.”^32^ Abramson’s concern about increasing IST commitments would prove prescient—50 years after the publication of his paper, the use of the IST commitment to institutionalize people with psychotic symptoms would be described as a national crisis.^29^

Six years later, in 1978, a Berkeley public policy student named Larry Sosowsky published a paper in *The American Journal of Psychiatry.* This study evaluated arrests for a cohort of 301 state hospital patients for 3 ½ years prior to LPS and 4 ½ years post LPS. The author documented a marked increase in arrests for this population post LPS implementation. He also looked at post-LPS arrests rates for the cohort compared to the general population and was able to demonstrate that the state hospital cohort was nine times more likely to be arrested than the general population.^33^

An April 15, 1979, story in the *Washington Post* noted that in California, “Crowding within the state’s financially strapped system has grown to the point where some mental patients, unable to find a hospital bed, are being arrested and placed in county jails for their own safety. Some have spent as long as a month in prison waiting for a mental hospital bed […] At the Los Angeles County jail there are sometimes as many as 50 mentally disabled persons locked up as long as a month waiting for a bed in the state hospital system.”^34^

In 1988, Thomas Arvanites of Villanova University published a paper in the journal *Criminology* looking at IST populations across three states. IST commitments increased post-deinstitutionalization by an average of 20%. The increase in IST admissions, as a percentage of all hospitalizations, was positively correlated to the rate of deinstitutionalization (r = 0.93). Arvanites noted, “An examination of the nature and operation of an IST commitment reveals its potential to emerge as an alternative to civil hospitalization.”^35^

In 2010, E. Fuller Torrey provided a review of many of the studies documenting the recriminalization of psychotic illness. Torrey conducted research concluding that there were three times more seriously mentally ill persons in jails and prisons than in hospitals. This work led to the oft-cited observation that the institutions housing the most patients with serious mental illness (SMI) in the United States were Riker’s Island jail in New York City, Cooks County jail in Chicago, and Twin Towers jail in Los Angeles. Torrey concluded, “It is thus fact, not hyperbole, that America’s jails and prisons have become our new mental hospitals.”^36^

In a 2018 retrospective analysis by the National Association of State Mental Health Program Directors Research Institute, Amanda Wik et al. documented a 72% increase in IST patients in state hospitals from 1999 to 2014.^30^ A separate survey conducted around this time identified that 82% of the states were experiencing a recent surge in IST patients, with a significant percentage facing litigation due to an inability to admit this increasing population in a timely fashion. In this survey, the reasons identified by the states centered on a lack of community mental health services as the primary driver of the crisis in their state.^29^ Research conducted by Barbara McDermott at the University of California Davis demonstrated that the majority of IST patients had schizophrenia spectrum disorders. Fully 47% were completely unsheltered, living on the open streets, and two thirds were experiencing some type of homelessness at the time of their arrest on felony charges. Half had received no mental health services prior to their arrest, and those who did were largely seen in emergency settings.^37^

The IST crisis has produced a cyclical pattern of blame. Federal lawsuits blame states for failing to provide timely admission to swelling populations of patients found IST. States blame local authorities for failing to adequately care for these patients in the community. Local authorities blame a lack of federal reimbursement to provide the type of care needed to wrap around and house these complex patients.

The IST crisis illustrates the consequences of failing to hold systems accountable for the care of people living with schizophrenia. Conditions have reverted to those found by Dorothea Dix 200 years ago—people with psychotic illness are kept in cages (in the form of jail cells) or left on the streets to die. They have once again been abandoned by society. Rather than achieving the desired goal of true deinstitutionalization, the lack of fiscal accountability inherent in the deinstitutionalization movement has instead resulted in the reinstitutionalization of people with psychotic illnesses in jails and forensic hospitals, through a circuit of homelessness, neglect, and criminal justice involvement. The civil liberties protection envisioned by the dangerousness standard has enabled the mental health system to turn away these patients who do not know that they are ill^38^^-^^40^ and who are often homeless, hungry, and tortured by their symptoms because “they don’t want treatment” and they are not evidencing the extreme dangerousness made necessary by the LPS act five decades ago.

## Italy as a model

The wheels of policy reform are starting to turn again. This time, there is an existing model providing hope. Reforms in Italy have succeeded proving that when systems are held accountable, people living with psychotic illness can lead meaningful lives in their community. Italy has all but eliminated both the institutionalization and criminalization of schizophrenia, through accountability in community funding combined with the elimination of the dangerousness standard for treating people who lack medical decision-making capacity.^41^^,^ ^42^ Italian psychiatrists have reported that, “Policymakers and healthcare providers should note that closing psychiatric hospitals was not the main objective of the Italian reform; rather, it was to ensure that citizens with mental disorders would be treated just as other patients. This principle has revolutionized the role and focus of psychiatry in Italy, from custody, coercion, and segregation to treatment and care.”^41^

Contrast this with the United States, where fractured funding and inadequate infrastructure, combined with a lack of accountability on systems for poor outcomes, are undoubtedly the primary culprits in the current crisis. The all-too-common outcomes of homelessness, incarceration, forensic institutionalization, and early death are enabled by the impossibility of obtaining informed consent from someone who does not understand that they are ill. The requirement that a person who lacks medical decision-making capacity must become dangerous in order to receive evidence-based care results in trauma, victimization, homelessness, brain damage, incarceration, and death. In other words, current civil liberties protections unintentionally provide a shield from accountability for failing to treat the sickest patients in our societypeople who do not comprehend that they are ill and who are prisoners of their own psychotic thoughts. Italy has demonstrated that aligning informed consent practices to those used for every other medical condition, in concert with adequately funding a community mental health system, works. Italian policies have resulted in vastly decreased involuntary commitments, nominal numbers of people with SMI in carceral settings, and the successful closure of all state and forensic hospitals nationwide.^42^

## Conclusion

To catch up with more advanced nations, the United States needs to delineate systems, policies, measures, and funding mechanisms specifically for schizophrenia spectrum disorders. Grouping psychotic disorders under the larger SMI umbrella enables systems to focus on other disease states to the exclusion of psychotic illness.

Public policy reform must eliminate funding incentives that reward the neglect of people living with psychotic disorders. One option is the creation of funding mechanisms that force the same system that fails patients in the community to pay for the much more expensive institutional care. Accountability can be baked into funding streams by tracking the primary outcomes of arrest, homelessness, incarceration, institutionalization, and death, and predicating funding on performance against these outcomes.

US reforms have started in to align the informed consent process for schizophrenia with the process for every other disease, specifically by utilizing the standard medical decision-making model.^43^ This prevents the current paradox of trying to obtain informed consent from a person who lacks medical decision-making capacity, and denying treatment when this impossible task is not accomplished.

Reforms should provide targeted, specific funding for a continuum of care for schizophrenia spectrum disorders, including early intervention, family psychoeducation and support, assertive community treatment, robust psychopharmacology, vocational and peer support, and a housing continuum that includes staffed supportive housing. These services should be individualized, and trauma informed.

Both the asylum and deinstitutionalization movements were born of noble intentions, and both failed in implementation in the context of inadequate funding and a lack of accountability. As the horrific conditions on our streets and in our carceral settings begin to drive change, there is no excuse to fail again.
